# Defence systems encoded by core genomic islands of seventh pandemic *Vibrio cholerae*

**DOI:** 10.1098/rstb.2024.0083

**Published:** 2025-09-04

**Authors:** Melanie Blokesch

**Affiliations:** ^1^ Laboratory of Molecular Microbiology, Global Health Institute, School of Life Sciences, Ecole Polytechnique Federale de Lausanne (EPFL), Lausanne, Switzerland

**Keywords:** defence systems, pandemic *V. cholerae*, mobile genetic elements, phages, plasmids

## Abstract

*Vibrio cholerae,* the causative agent of cholera, has triggered seven pandemics, with the seventh pandemic emerging in 1961. The success of seventh pandemic El Tor (7PET) *V. cholerae* as a human pathogen is linked to its acquisition of mobile genetic elements (MGEs) like the CTXΦ prophage and *Vibrio* pathogenicity island 1 (VPI-1). Additional MGEs, including VPI-2 and the *Vibrio* seventh pandemic islands (VSP-I and VSP-II), are thought to have further enhanced the pathogen’s virulence potential. However, recent research suggests that these MGEs serve as reservoirs for defence systems, which may represent their main role. In this review, I highlight conserved defence systems in the 7PET lineage, including a cyclic-oligonucleotide-based antiphage signalling system, DNA defence modules (DdmABC and DdmDE), an anti-viral cytidine deaminase, and diverse restriction systems (T1RM and TgvAB). I also discuss two defence systems encoded on a VSP-II variant unique to West Africa–South America lineage strains and the chromosomal integron, recently recognized as a biobank for defence systems. Lastly, I address the challenges posed by the scarcity of *V. cholerae* phages, which often require studying these systems in heterologous hosts like *Escherichia coli*, leaving their natural triggers and defence roles against predatory phages of 7PET *V. cholerae* largely unexplored.

This article is part of the discussion meeting issue ‘The ecology and evolution of bacterial immune systems’.

## 1. Introduction

*Vibrio cholerae*, the causative agent of cholera, has triggered seven pandemics over the past two centuries. However, evidence suggests that cholera is an ancient disease, with descriptions of its symptoms appearing in Sanskrit writings as early as the fifth century BC [1]. The seventh pandemic began in 1961 in Indonesia and has since spread globally [1]. Unlike the *V. cholerae* O1 classical strains responsible for the fifth and sixth cholera pandemic—and probably earlier ones—the current pandemic is caused by *V. cholerae* O1 serogroup isolates known as the seventh pandemic El Tor (7PET) strains [2]. Over the past six decades, these strains have spread to multiple countries and continents, primarily originating from cholera-endemic regions in Asia, such as Bangladesh and eastern India around the Bay of Bengal [3]. These transmission events occurred in three major waves, with wave 1 including further spread from West Africa to South America in the 1990s. Consistently, the *V. cholerae* isolates associated with this event, which include widely used model strains such as A1552, C6706 and C6709, were named the West Africa–South America (WASA) lineage [3].

The ability of *V. cholerae* to cause disease relies on two primary virulence factors [4]. The first is the cholera toxin (CT) [5], an AB5-type toxin that binds to GM1 gangliosides on the epithelial cells of the small intestine and enters these cells through retrograde trafficking [6,7]. Once inside, CT disrupts the intracellular cAMP balance, leading to the activation of chloride channels. This disruption causes an ion imbalance and the loss of large amounts of water, manifesting as the severe diarrhoea characteristic of cholera [1].

The second major virulence factor, the toxin-coregulated pilus (TCP), is co-regulated with CT, which accounts for its name [8]. TCP is a type IV pilus and essential for intestinal colonization, as it enables the formation of microcolonies on the surface of intestinal cells thereby enhancing the pathogen’s ability to establish and sustain infection [8,9].

**Figure 1.**
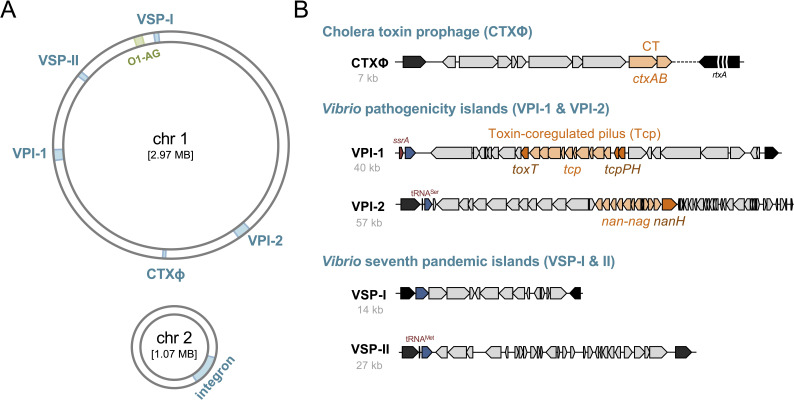
Core mobile genetic elements (MGEs) in pandemic *V. cholerae*. (A) Overview of the two chromosomes of *V. cholerae*, indicating the locations of core MGEs associated with 7PET strains and the integron on chromosome 2. (B) Schematic representation of the core MGEs: CTXɸ, VPI-1, VPI-2, VSP-I and VSP-II. Virulence-related genes are highlighted in orange. The size of each MGE is noted below its name. The diagrams are not drawn to scale but are optimized for clarity and visualization. Details on these genomic islands, including new discoveries revealing that they encode several defence systems, are presented in the following figures. To ensure a consistent depiction of the genomic islands, all are shown with the integrase gene aligned to the left.

Both CT and TCP are encoded on mobile genetic elements (MGEs). Precisely, the genes encoding CT are carried by the CTXΦ prophage [10], which integrates into either the large or small chromosome of *V. cholerae* ([3,10–13]; figure 1). Interestingly, the acquisition of CTXΦ relies primarily on TCP as a receptor for cellular entry [10]. The *tcp* operon, in turn, is located on the *Vibrio* pathogenicity island 1 (VPI-1; [14,15]), which also encodes key virulence regulators such as TcpP/H and ToxT ([16]; figure 1). Despite initial claims suggesting that VPI-1 might also constitute a prophage [17], subsequent studies did not support this hypothesis [18–20], and no further investigations on this aspect have been published since 2003. Nonetheless, it is well-established that the acquisition of these two MGEs—the CTXΦ prophage and VPI-1—was critical in transforming *V. cholerae* from a common aquatic bacterium into a major human pathogen [19].

**Figure 2.**
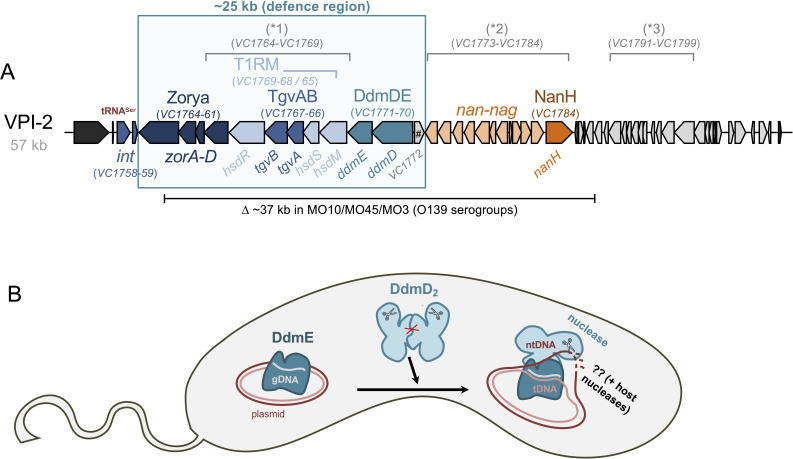
VPI-2-encoded defence systems. (A) Schematic representation of VPI-2, highlighting its three initially described regions (*1, *2, *3) and the corresponding locus tag numbers based on reference strain N16961. The approximately 37 kb region deleted (∆) in O139 serogroup-converted 7PET strains is shown below the schematic. The region referred to as the ‘defence region’ is shown in the box. Genes encoding the four defence systems—Zorya, T1RM, TgvAB and DdmDE—are highlighted in different colours, with their respective genes indicated. The *nan-nag* and *nanH* genes, encoding functions related to sialic acid uptake and catabolism and a neuraminidase/sialidase, respectively, are also shown. *int*, integrase genes. (B) Model of plasmid elimination by the DdmDE system. DdmE, a prokaryotic argonaute-like protein, binds single-stranded DNA guides (gDNA) to identify its plasmid targets (tDNA). It then recruits the effector protein DdmD, which is typically kept in an autoinhibited dimeric state (DdmD_2_), as indicated by the red cross. Upon loading onto the non-target DNA strand (ntDNA), DdmD’s helicase activity enables translocation along the ssDNA, ultimately resulting in DNA cleavage in a nuclease domain-dependent manner. Whether additional host nucleases are required for the complete elimination of the plasmid remains unclear.

Additional MGEs have been identified in pandemic *V. cholerae*, including *Vibrio* pathogenicity island 2 (VPI-2; [21]) and the two *Vibrio* seventh pandemic islands (VSP-I and VSP-II), which are hallmark features of 7PET strains ([22]; figure 1). Compared to the well-characterized CTXΦ prophage and VPI-1, the functions of these additional MGEs have remained less understood, with some exceptions. For instance, VPI-2 encodes a neuraminidase/sialidase (NanH; figure 1), which subtly enhances CT binding and uptake by intestinal cells [23], probably by cleaving sialic acid to expose the GM1 gangliosides on the intestinal surface [24]. Moreover, the freed sialic acids (*N*-acetylneuraminic acid) can serve as a carbon source for *V. cholerae* [24]. In addition, VPI-2 encodes enzymes for sialic acid uptake and catabolism (*nan-nag* genes; figure 1), which confer a competitive colonization advantage in infant mice [25]. Nonetheless, most *V. cholerae* O139 strains (such as reference strains MO10 and MO45 [26])—serogroup-converted derivatives of the 7PET lineage [27–29] and identified as causative agents of cholera during the 1990s—lack the *nan-nag* region and *nanH* ([24]; figure 2). This observation suggests that these parts of VPI-2 are not essential for cholera pathogenesis. Interestingly, the *nan-nag* region (with or without *nanH*) is also widely distributed among non-pandemic *V. cholerae* strains and even environmental isolates [30] as well as in *Vibrio mimicus* [31], indicating that this cluster may serve functions beyond the human host.

Consistently, recent research has provided evidence that VPI-2 and the other genomic islands, along with additional MGEs, may not, or not solely, contribute directly to pathogenesis. Instead, they appear to play an important role in bacterial defence against MGEs such as plasmids and bacteriophages, thereby indirectly influencing transmission dynamics. Indeed, a defining characteristic of pandemic *V. cholerae* is the presence of their MGEs [19]. As outlined earlier, the primary MGEs in 7PET strains include CTXΦ, VPI-1, VPI-2, VSP-I and VSP-II. Since CTXΦ and VPI-1 are not known to encode defence systems, this review will focus on the remaining three core genomic islands—VPI-2, VSP-I and VSP-II—of 7PET *V. cholerae*, while also highlighting recent research related to the integron island. Moreover, the accompanying article by Blokesch & Seed [32] reviews accessory MGEs of sixth pandemic classical and 7PET strains, including phage-inducible chromosomal island-like elements (PLE), integrative and conjugative elements (ICEs), and the WASA-1 prophage, as well as their defence functions in the context of the ecology and evolution of pandemic *V. cholerae* [32]*.*

Note that, this review refers to locus tag numbers according to the reference strain N16961 [33], where applicable, but provides a map based on a newly sequenced and *de novo* assembled genome of this strain [34] in figure 1, reflecting recent findings of a misassembly in the initial genome map [35,36].

## 2. *Vibrio* pathogenicity island 2

The VPI-2 island, which harbours 52 open reading frames (ORFs), was first identified in 2002 by the Boyd laboratory based on its lower GC content (42% compared to 47% in the chromosomal backbone), the presence of two integrase genes, and its insertion into a transfer RNA locus (tRNA^Ser^) [21]. The genetic organization of the 57.3 kb VPI-2 island was initially divided into three major regions (figure 2A): (i) a cluster of five genes predicted to encode a type I restriction–modification system; (ii) the *nan-nag* region; and (iii) a phage-like region of unknown function [21]. Variations of VPI-2, which suggest a mosaic composition of the island, have been identified in non-7PET *V. cholerae* as well as in *V. mimicus*, indicating the island’s horizontal transfer [30,31].

The initial characterization of VPI-2 primarily focussed on the sialic acid utilization cluster (*nan-nag*) and *nanH*, as mentioned above [24]. However, recent research suggests that VPI-2 may function as a *bona fide* defence island. Defence islands were first described by Makarova *et al*., who observed that anti-phage defence systems and mobile genes frequently cluster together within genomic islands [37]. Notably, their analysis identified gene families that were over-represented in these defence islands, suggesting that they might constitute novel defence systems. This ‘guilt-by-association’ approach was later expanded by Doron *et al.* from the Sorek laboratory, who subsequently validated several newly predicted defence systems experimentally through artifical expression in *Escherichia coli* and *Bacillus subtilis* [38]. In the case of VPI-2 in 7PET *V. cholerae*, four defence systems have independently been identified within the approximately 25 kb region downstream of the integrase genes (figure 2A). Of these, three—namely, the type I restriction–modification (T1RM) system, type I-embedded GmrSD-like system of VPI-2 (TgvAB) and DdmDE—have been experimentally verified [39–41]. I refer to this approximately 25 kb segment as the ‘defence region’ of VPI-2 throughout this text. Notably, *V. mimicus* lacks this portion of VPI-2 [31].

### (a) Type I restriction–modification system

The T1RM and TgvAB gene clusters are part of the originally described five-gene type I restriction-modification region of VPI-2 [21]. Like other type I RM systems, which catalyse both restriction and modification [42], the VPI-2-encoded T1RM system consists of three subunits: the methylase HsdM (VC1769), the specificity subunit HsdS (VC1768), and the restriction enzyme subunit HsdR (VC1765). Recent research by our laboratory has demonstrated that the T1RM system is active in 7PET *V. cholerae*, methylating the genome at approximately 600 copies of the GATGNNNNNNCTT motif (*m6A:**GATGNNNNNNCTT**:2*) [40]. By contrast, the O139 serogroup strain MO10, which lacks this region of VPI-2 (figure 2A), was found to be non-methylated at this motif. Similarly, a recent African 7PET isolate was shown to lack methylation owing to an *IS256* insertion sequence within *hsdS* [40], a phenomenon also observed in other isolates from the Democratic Republic of the Congo (DRC) [43]. However, these and other strains possess alternative or additional epigenetic marks encoded by their SXT-like ICEs. Specifically, *m6A:GTTRAAG:6* marks are associated with SXT/*Vch*Ind4-harbouring isolates such as O139 strains MO10, MO45 and MO3, while *m6A:RTAAAYG:5* marks are found in *Vch*Ind5-carrying strains [44] like DRC193A [43] (M. Blokesch , unpublished data).

Importantly, through investigations in its native *V. cholerae* host, it was demonstrated that the VPI-2 T1RM system restricts incoming plasmids carrying the identified motif [40]. Phage defence by this T1RM system could not be observed in *V. cholerae*, as most *V. cholerae* vibriophages—such as the prominent International Centre for Diarrhoeal Disease Research phages (ICP) ICP1, ICP2 and ICP3 phages [45,46] (see accompanying article by Blokesch & Seed for details on ICP phages [32])—were isolated on VPI-2-positive pandemic 7PET or classical strains, excluding phages sensitive to this system. However, Gomez & Waters reported that when expressed from a plasmid, the T1RM gene cluster conferred strong protection to the heterologous host *E. coli* against diverse coliphages, including T3, λ, SECΦ18 and SECΦ27 [41].

### (b) Type I-embedded GmrSD-like system of VPI-2 (TgvAB)

Embedded within the T1RM gene cluster is a two-gene operon named *tgvAB* ([40,41]; figure 2A). Bioinformatic analysis of the TgvAB system revealed that TgvA (VC1767) and TgvB (VC1766) both possess an N-terminal DUF262 domain, while TgvB additionally contains a C-terminal DUF1524 domain. Interestingly, the GmrSD family of type IV restriction enzymes is known to harbour DUF262 and DUF1524 domains [47].

Structural modelling of TgvAB using AlphaFold [48] demonstrated that TgvB shares its domain architecture with Eco94GmrSD from *E. coli* strain STEC_94C [49], whereas for TgvA, this similarity is restricted to the N-terminal DUF262 domain [40]. Furthermore, TgvB models closely aligned with BrxU and SspE [50,51], providing additional evidence that it functions as a type IV DNA modification-dependent restriction enzyme [40]. For BrxU, the N-terminal DUF262 domain has been shown to play a role in nucleotide binding and hydrolysis. The current model therefore suggests that BrxU transitions from a dimeric to a monomeric state upon nucleoside triphosphate (NTP) binding, while reverting back to the dimeric state following NTP hydrolysis, potentially in concert with the recognition of modified DNA, followed by its cleavage [50]. However, the exact mechanism by which modified DNA is recognized by GmrSD-like enzymes remains unknown to date.

Production of the TgvAB system in *E. coli* provided evidence for robust phage protection [40,41]. Specifically, when screened against the BASEL phage collection of *E. coli* phages [52], the expression of *tgvAB in cis* led to a highly significant reduction in the efficiency of plaquing for all 15 tested members of the *Tevenvirinae* subfamily, including phages T2, T4 and T6 [40]. Indeed, Vizzarro *et al.* observed defence specifically against the *Tequatrovirus* and *Mosigvirus* groups, which represent the *Tevenvirinae* within the BASEL collection. Notably, both groups are characterized by the presence of modified cytosines. The *Tequatrovirus* phages contain glucosylated hydroxymethyl cytosines, while *Mosigvirus* phages possess arabinosylated hydroxymethyl cytosines [52]. The finding that T-even phages T2, T4 and T6 were restricted by TgvAB was corroborated by a concurrent study [41]. These authors additionally isolated T2 escape mutants that had lost the ability to glucosylate their cytosine residues, rendering them resistant to TgvAB [41]. Collectively, these data provide evidence that the system functions as a type IV DNA modification-dependent restriction enzyme, leading to its designation as the type I-embedded GmrSD-like system of VPI-2, or TgvAB [40,41].

### (c) DNA defence module DE

Two distinct plasmid defence systems conserved within the 7PET lineage were recently identified and characterized by our lab, which have been designated as DNA defence modules (Ddm) ABC and DdmDE [39]. DdmABC is encoded on VSP-II (see below), while the *ddmDE* operon is part of the approximately 25 kb ‘defence region’ of VPI-2 ([39]; figure 2A). It was demonstrated that these two systems collaborate within 7PET *V. cholerae* to swiftly eliminate plasmids [39,53]. Consistent with these findings—and in contrast to other *Vibrio* species and environmental *V. cholerae* strains [54,55]—7PET isolates are devoid of small plasmids and only sporadically carry large IncA/C-type plasmids [56,57].

The DdmDE system primarily targets small plasmids, with some exceptions and comprises two protein components (figure 2B). The first, DdmD, features an N-terminal superfamily 2 helicase domain with conserved residues essential for nucleotide binding and hydrolysis. Additionally, DdmD contains a C-terminal PD-(D/E)XK-superfamily nuclease domain, which is associated with DNA cleavage [39]. By contrast, no discernible domains were initially identified for DdmE. However, structural modelling suggested a potential connection between DdmE and prokaryotic Argonaute (pAgo) proteins, implying a possible functional relationship [39].

Follow-up studies aimed at elucidating the detailed mechanistic aspects of the DdmDE system through biochemical and structural characterization confirmed that DdmE belongs to the family of long pAgo proteins [53]. Cryo-electron microscopy (CryoEM) structures revealed that DdmE, as a catalytically inactive pAgo, employs DNA guides to target plasmid DNA, subsequently recruiting its partner protein, DdmD ([53,58–61]; figure 2B). Upon binding by DdmE, the autoinhibited dimeric architecture of DdmD disassembles, allowing DdmD to load onto the non-target strand of the plasmid DNA ([53,58–61]; figure 2B). Furthermore, *in vitro* studies revealed that DdmD facilitates single-stranded DNA (ssDNA) translocation in a 5′-to-3′ direction via a distinctive ‘gate-clamp’ mechanism, driven by its helicase domain, while partially degrading plasmid DNA through its nuclease domain [53,58–62]. Collectively, these studies confirmed the role of the DdmDE system as a defence mechanism against plasmids, with DdmE acting as a DNA-guided sensor protein that specifically targets plasmids and recruits the effector protein DdmD. Once recruited, DdmD translocates along the split plasmid DNA, ultimately leading to its cleavage in a nuclease domain-dependent manner (figure 2B). Whether DdmDE fully degrades the plasmid, merely nicks the DNA, or creates ssDNA gaps that may facilitate further degradation by host nucleases remains unresolved to this day.

BLAST analysis of the protein encoded upstream of the *ddmDE* operon (VC1772) suggests a regulatory role, given its helix-turn-helix and WYL (pfam13280) domains (figure 2A). The WYL domain, in particular, may function as a ligand-sensing domain [63]. This is consistent with previous observations of the ssDNA-binding ability of diverse WYL-domain proteins [64–66] and the role of WYL domain-containing transcription factors in regulating bacterial defence systems [67–69]. The potential role of VC1772 in regulating the *ddmDE* operon or other *V. cholerae* genes is currently being investigated in our laboratory.

### (d) A predicted Zorya system

Another part of the ‘defence region’ of VPI-2 encodes a system known as Zorya ([38]; figure 2A). While early studies noted similarities between two Zorya proteins, ZorA and ZorB, and the bacterial flagellar motor proteins MotA and MotB [38], a recent investigation has elucidated the detailed molecular mechanism of an *E. coli* Zorya system [70]. In brief, this Zorya type I system relies on the proton-driven, membrane-bound motor proteins ZorAB, which are activated upon phage infection. This activation signal is transmitted via the long cytosolic tail of ZorA to the effector proteins ZorC and ZorD, which subsequently degrade the phage DNA. Thus, Zorya functions as a direct phage defence system by eliminating the phage’s genetic material [70]. To our knowledge, the functionality of the *V. cholerae* 7PET Zorya homologue (figure 2) has not been demonstrated yet—either in *V. cholerae* or in the heterologous host *E. coli*. Indeed, our attempts to demonstrate the system’s activity have so far been unsuccessful, suggesting that further optimization of experimental conditions may be necessary in the future.

## 3. *Vibrio* seventh pandemic island I

Following the elucidation of the genome sequence of the 7PET strain N16961 [33], genomic hybridization studies using microarrays enabled the first comparative genomic analyses of diverse *V. cholerae* strains [22]. Through these analyses, genes unique to 7PET strains—absent from sixth pandemic classical strains and pre-seventh pandemic O1 El Tor strains, such as the commonly referenced MAK757 strain—were identified in 2002 by Dziejman *et al* [22]*.* The majority of these 7PET-specific genes were found to belong to two genomic regions, named *Vibrio* seventh pandemic island I (VSP-I) and II (VSP-II) (figure 1). Importantly, high-resolution comparative genomic analysis of strains collected between 1930 and 1964—spanning the evolution from the first available El Tor biotype strain to the onset of the seventh pandemic—concluded that the incorporation of VSP-I and VSP-II, along with an El Tor-type version of CTXΦ, played a crucial role in the transition of pre-pandemic El Tor strains into the broadly transmitted 7PET lineage [13].

**Figure 3.**
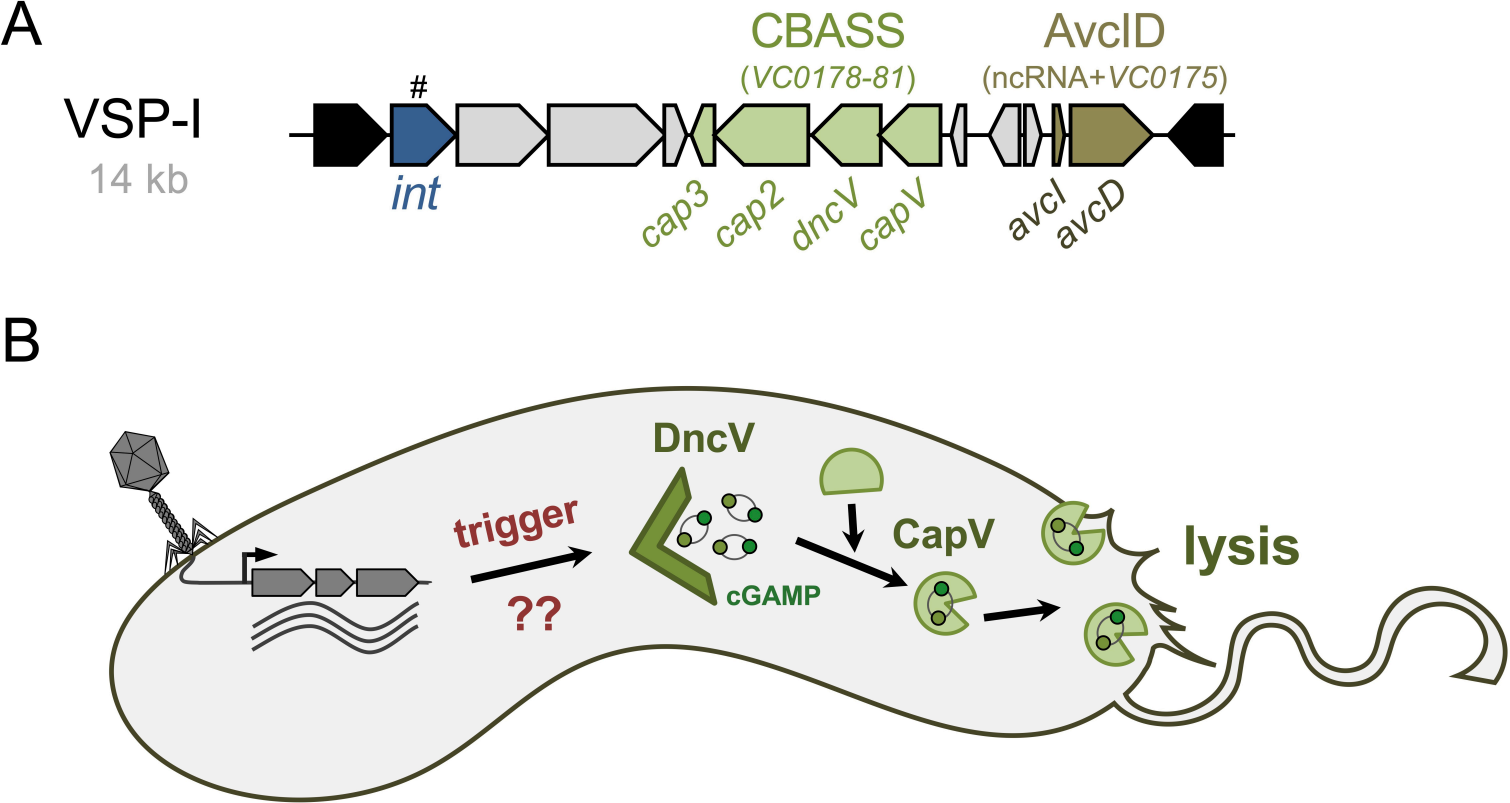
VSP-I-encoded defence systems. (A) Schematic representation of VSP-I. Genes encoding the two defence systems—CBASS and AvcID—are highlighted in different colours, with their respective genes indicated. ncRNA denotes non-coding RNA AvcI. The integrase gene (*int*), marked with a hash (#) symbol, is interrupted by *ISVch4*-family transposase genes in the WASA lineage of 7PET strains. (B) Simplified model of the CBASS system. Upon an unidentified trigger following phage infection, DncV is thought to synthesize cGAMP, which subsequently binds to and activates the effector protein CapV. The phospholipase CapV degrades phospholipids in the cell membrane, leading to cell death and aborting phage replication. For simplicity, the Cap2-mediated activation of DncV through C-terminal target conjugation and the cleavage of such DncV–target conjugates by Cap3 are not shown.

The 14 kb VSP-I spans genes VC0175 to VC0185 (figure 3A). The genes in this genomic island were initially classified as encoding hypothetical or conserved hypothetical proteins, with a few exceptions. VC0185 was putatively annotated as a transposase, VC0175 as a deoxycytidylate deaminase-related protein, VC0176 as a helix-turn-helix motif-containing transcriptional regulator, and VC0178 as a phospholipase based on its homology to such enzymes [22]. However, their biological functions remained largely unknown for more than a decade.

### (a) Cyclic-oligonucleotide-based antiphage signalling system

Work by the Mekalanos laboratory in 2012 elucidated the first known function of VSP-I. They showed that the gene *VC0179* encodes a dinucleotide cyclase (DncV; figure 3A), which synthesizes a previously undescribed hybrid cyclic AMP-GMP molecule, 3′,3′-cyclic GMP-AMP (cGAMP) [71]. Interestingly, in its 2′,3′ conformation, cGAMP functions as a primary innate immune signalling molecule in eukaryotes [72,73]. Consistent with this, DncV was identified as a structural and functional homologue of human cyclic guanosine monophosphate–adenosine monophosphate synthase (cGAS), an immune sensor of cytosolic DNA that triggers inflammation [72]. Research has since revealed that cGAS-like enzymes, also known as cGAS/DncV-like nucleotidyltransferases (CD-NTases), are capable of synthesizing noncanonical signals, including cyclic dinucleotides, cyclic trinucleotides and linear oligonucleotides, and are widely distributed in bacteria [74].

DncV was initially reported to be required for efficient intestinal colonization of *V. cholerae* [71]. However, more recent research indicated that neither VSP-I nor VSP-II contribute to intestinal colonization in an infant mouse model of cholera [75], suggesting that their primary role may lie outside of pathogenesis. Supporting this notion, the Sorek laboratory demonstrated in 2019 that the *dncV*-containing four-gene operon in VSP-I of *V. cholerae* constitutes a phage defence system when expressed in *E. coli* ([76]; figure 3B). Upon phage infection, DncV synthesizes cGAMP, which activates a phospholipase, as previously described [77]. This activation triggers membrane degradation and ultimately results in cell death, serving as a form of abortive infection ([76–78]; figure 3B). The cGAMP-dependent activation mechanism forms the foundation of the system, which was subsequently named the cyclic-oligonucleotide-based antiphage signalling system (CBASS) [76].

The last two genes in the four-gene operon encode CD-NTase-associated proteins 2 (Cap2) and 3 (Cap3) (figure 3A), which show homology to ubiquitin-conjugating (E1/E2) and deconjugating enzymes, respectively [79]. Cap2 modifies the C-terminus of DncV by ligating it to unidentified target proteins *in vivo.* Cap3, a specific endopeptidase, regulates Cap2 activity by cleaving CD-NTase–target conjugates [79,80].

As mentioned above, these enzymes share an evolutionary ancestry with the cGAS–STING innate immune pathway of animals [81–84]. However, bacterial CD-NTases differ from their eukaryotic counterparts in several ways. They do not respond to nucleic acids *in vitro* [74], and the absence of spatial compartmentalization in bacteria suggests that CBASS immunity does not function through the recognition of mislocalized DNA [85]. Moreover, many bacterial CD-NTases are inherently active *in vitro* without the need for an activating ligand [74]. This evidence supports a model where CBASS immune systems are maintained in an inactive state by inhibitory molecules, which repress CD-NTase activation in the absence of phage infection.

Identifying the precise activating cue that triggers DncV signalling in *V. cholerae* remains a significant unresolved question. However, recent research has shown that folates inhibit the cyclase activity of CD-NTases, and that folate synthesis inhibitors, such as sulfonamide and trimethoprim, lead to increased cGAMP production in DncV-producing *E. coli* [86]. This observation was further validated in *V. cholerae*, where the use of folate biosynthesis inhibitors (e.g., sulfamethoxazole, SMX) led to increased cGAMP production when the bacteria were grown to high cell density [75] and to bacterial lysis caused by these otherwise bacteriostatic antifolate antibiotics [87]. Consistently, VSP-I-encoded CBASS-carrying 7PET strains exhibited greater sensitivity to antifolate antibiotics compared to classical strains or CBASS deletion mutants [75,87]. Furthermore, exposure to antifolates *in vitro* led to the emergence of 7PET suppressors with inactivated CBASS systems [75,87]. Based on these findings, it has been suggested that folate depletion caused by phage infection may serve as a native cue for CBASS activation and subsequent bacterial cell death in 7PET strains [75,87].

Collectively, the functionality of the VSP-I-encoded CBASS as a *bona fide* phage defence system has only been demonstrated in heterologous hosts. Future research is required to investigate this system in its native host under physiological, non-overexpression conditions, using native vibriophages against which the system provides defence.

### (b) Anti-viral cytidine deaminase

A second phage defence system encoded on VSP-I, named anti-viral cytidine deaminase (AvcD), was first described in 2022 by the Waters laboratory [88]. The activity of this enzyme is post-translationally inhibited by a small RNA, AvcI, encoded adjacent to *avcD* (figure 3A). Together, AvcID constitutes a toxin–antitoxin (TA) system that functions as a phage defence mechanism. It was initially proposed that upon infection, transcription inhibition reduces the levels of the AvcI sRNA, similar to type III TA system toxins such as ToxIN [89]. This loss of AvcI releases the AvcD enzyme from inhibition. Subsequently, AvcD reduces the availability of free deoxycytidine nucleotides, thereby halting phage replication [88].

In phage infection experiments performed in the heterologous host *E. coli*, where the *avcID* operon was expressed from a plasmid, weak protection against phage T3 was observed for the *avcID* system from *V. cholerae*. By contrast, the *Vibrio parahaemolyticus* homologue provided robust protection against several coliphages [88]. Using this experimental set-up of expressing the *V. parahaemolyticus avcID* system from a medium-copy-number plasmid in *E. coli*, follow-up work confirmed that the loss of AvcI occurs following inhibition of host cell transcription, leading to activation of AvcD. Furthermore, the authors demonstrated that both coliphages, T5 and T7, caused a decrease in AvcI levels and, consequently, a reduction in intracellular dCTP and dCMP, showing that both phages activate AvcD [90]. However, the system only protected bacteria against T5 and not T7, which the authors attributed to the faster replication cycle of T7, probably enabling sufficient genome replication before dCTP depletion becomes critical. These studies, along with concurrent work by the Sorek laboratory, demonstrated that antiviral nucleotide-targeting defence systems are widespread across numerous bacterial species [88,90,91].

## 4. *Vibrio* seventh pandemic island II

**Figure 4.**
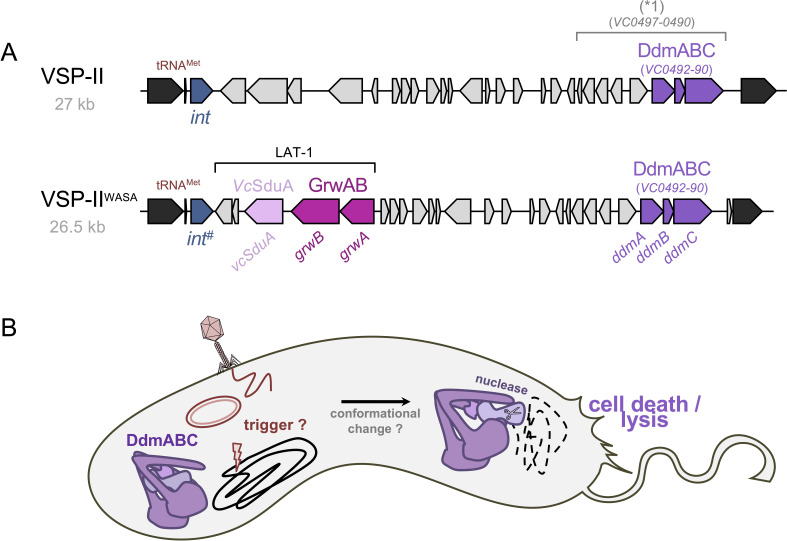
VSP-II-encoded defence systems. (A) Schematic representation of VSP-II, highlighting the initially described region (*1) and the corresponding locus tag numbers based on reference strain N16961. The lower section illustrates the VSP-II variant specific to the WASA lineage of 7PET *V. cholerae*, where the ‘LAT-1 region’ replaces the original genes *VC0511–VC0515* of VSP-II. Note that, the integrase gene also differs compared to the reference gene *VC0516,* retaining only 89.5% nucleotide identity (indicated by int^#^). Genes encoding the three defence systems—*Vc*SduA, GrwAB and DdmABC —are highlighted in different colours, with their respective genes indicated. (B) Model of DdmABC’s anti-plasmid and anti-phage activity. Upon an unknown trigger—arising from phage and/or plasmid replication, DNA stem loops, DNA damage or stalled replication (the latter three symbolized by a flash)—the DdmABC system becomes activated. This activation probably induces a conformational change that releases the DdmA effector nuclease, which then cleaves DNA nonspecifically, including the bacterial chromosomal DNA. The strength of the trigger probably varies between plasmids and phages, with phage infection causing cell death thereby aborting phage replication.

VSP-II was initially defined as encompassing genes *VC0490–VC0497* (and potentially also *VC0498–VC0502*) ([22]; figure 4A). However, this definition was later revised to include a larger approximately 27 kb region, extending from *VC0490* to *VC0516*, as the canonical VSP-II (figure 4A ;[92]). This region includes genes that encode an integrase (e.g. *VC0516*), which facilitates sporadic excision of the island under laboratory conditions [93], as well as a ribonuclease, a type IV pilin, methyl-accepting chemotaxis proteins, and several transcription regulators [92].

Despite these findings, the functional roles of most VSP-II-encoded genes remained largely unknown until recently. Exceptions include the Zur-regulated VSP-II gene products, such as the peptidoglycan hydrolase ShyB (*VC0503*) [94], and the AraC-like transcriptional factor VerA, which activates the chemoreceptor gene *aerB* to promote oxygen-dependent congregation and energy taxis in zinc-starved environments [95].

### (a) DNA defence module ABC

Comparative genomic studies have indicated that several variants of VSP-II exist in diverse *V. cholerae* strains, with some lacking specific sub-modules of this island [96]. However, the part of the island encompassing genes *VC0492–VC0490* has remained relatively stable throughout the seventh pandemic [96], with the exception of some very recent Bangladeshi 7PET strains, isolated between 2018 and 2022, that have lost or mutated this region of VSP-II [97].

Work by our laboratory has demonstrated that the *VC0492–VC0490* operon encodes a dual-function plasmid and phage defence system, which was named the DNA defence module ABC or DdmABC ([39]; figure 4A). Analysis of the DdmABC system revealed that DdmC is an SMC-like protein featuring N- and C-terminal nucleotide-binding motifs, separated by coiled-coil arms. DdmA contains a Cap4 endonuclease domain and an unidentified C-terminal domain, while DdmB encodes a small protein with a DUF6521 domain (figure 4B). This configuration aligns with operons classified under a novel ABC-3C (for ABC-three component) clade with potential roles in defence [98]. These three-gene clusters, including *ddmABC*, were subsequently classified as Lamassu type II systems [99,100] owing to their distinctiveness from the originally identified two-gene Lamassu type I system [38]. The exact molecular interplay between the three subunits and the mechanisms governing the system’s activation state are currently under investigation.

The presence of certain plasmids has been shown to trigger DdmABC activity, resulting in plasmid-dependent toxicity in *V. cholerae* and the heterologous host *E. coli* ([39]; figure 4B). This activity imposes a fitness disadvantage on plasmid-carrying cells. Hence, in the absence of selective pressure, such as antibiotics that select for plasmid-encoded resistance genes, plasmids are rapidly lost from 7PET strains. Interestingly, comparative genomic analyses by Weill *et al*, particularly on 7PET isolates from Africa, revealed that large conjugative plasmids of the IncA/C group were highly abundant towards the end of wave 1 of the seventh pandemic but have since become rare [56]. Concomitant with the transition from wave 1 to wave 2, 7PET strains acquired ICEs that also conferred antibiotic resistance. It is tempting to speculate that the DdmABC system played a role in this shift, as ICE elements, unlike plasmids, replicate as part of the chromosome and are therefore probably invisible to this defence system [39].

When chromosomally expressed in *E. coli*, DdmABC also provides defence against phages P1 and λ through an abortive infection mechanism ([39]; figure 4B). This phage defence by the DdmABC system has recently also been confirmed in its natural host, *V. cholerae* [101–104]. Among these studies, DdmABC-dependent protection levels were reported to range from 10³- to 10⁴-fold against phage X29 [103,104], and up to >10⁷-fold for phage VIB04 [102]. Notably, protection against phage VIB04, previously known as vibriophage N4, was only abolished upon deletion of the entire VSP-II, whereas it remained fully intact in 16 site-directed *ddmABC* mutants [102]. Several of these mutants produced severely truncated proteins, lacking up to two-thirds of their sequence owing to nonsense or frameshift mutations, or gene interruptions. While the authors concluded that these severely altered DdmABC variants retained residual anti-phage activity, this interpretation should be viewed with caution [102].

In a separate study, O’Hara *et al* reported a comparatively modest 2.5-fold protection against a newly isolated ICP3 phage (ICP3_2016_M1; M1Ф). Notably, the loss of protection was only observed in strains lacking both AvcD and DdmC, while a *ddmABC* deletion exhibited a wild-type (WT)-like phenotype [101]. Interestingly, a phage variant isolated from the same cholera stool sample (ICP3_2016_M2; M2Ф), carrying an amino acid change in its DNA polymerase, exhibited complete resistance to AvcD and DdmABC [101]. This phage’s faster replication cycle [101] probably allowed sufficient genome duplication before dCTP depletion, matching the above-described impact of replication speed on AvcD activity [90]. It is tempting to speculate that AvcD-dependent dCTP depletion induces DNA damage, subsequently activating the DdmABC system independently of the initial phage insult. This hypothesis aligns with recent findings from the Mekalanos laboratory, which suggested that palindromic DNA sequences in plasmids or phages, or even UV-induced stress, might activate the DdmABC system through DNA damage and repair events ([102]; figure 4B). These authors also confirmed the previously reported plasmid-triggered cell death associated with DdmABC activity [39] when cells were grown to high density [102].

### (b) GmrSD-like type IV REase of West Africa–South America strains (GrwAB)

Comparative genomic studies have revealed a striking change in VSP-II, which occurred in the WASA lineage of wave 1 pandemic *V. cholerae* strains (figure 4A). In this lineage, VSP-II lacks several canonical genes (*VC0511–VC0515,* with the integrase gene *VC0516* being replaced by a homologous gene; figure 4A; [3,105]). Instead, these strains incorporated a novel module consisting of three genes and a frameshifted transposon, sometimes referred to as the Latin American transmission (LAT-1; [106]) change of VSP-II, which collectively encode two novel phage defence systems ([104]; figure 4A).

The first system, named GmrSD-like Type IV REase of WASA strains (GrwAB), contains two proteins with N-terminal DUF262 domains. However, while one of the proteins, GrwA, also features a DUF1524 domain, similar to TgvB described above, its partner protein, GrwB, differs from TgvA by containing multiple unknown domains in its extended C-terminus [104]. Notably, canonical GmrSD-like type IV restriction systems (e.g. GmrSD, BrxU, SspE; [49–51]) typically consist of single proteins. By contrast, TgvAB and GrwAB are unusual as they function as two-protein systems, requiring both partner proteins for activity. This difference warrants further investigation in the future.

Since no *V. cholerae* phages with modified genomes have been described to date, the defence capacity of the GrwAB system was tested by expressing it chromosomally in *E. coli* and using the BASEL phage collection to screen for protection [52]. Potent anti-phage activity was observed against all members of the *Tevenvirinae*, which possess glucosylated or arabinosylated hydroxymethyl cytosines in their genomes. Interestingly, the system also conferred protection against *Queuovirinae* phages, which feature 7-deazaguanine modifications [52,107]. Escape mutants of GrwAB-targeted phages were subsequently isolated, with mutations identified in the 7-deazaguanosine-inserting enzyme DpdA, supporting the hypothesis that DNA modifications are recognized by the system [104].

Collectively, these findings demonstrate that the VSP-II variant of the WASA 7PET lineage contains an additional type IV restriction system, GrwAB, which targets a broader range of phages than the VPI−2-encoded TgvAB system. Notably, the detailed molecular mechanisms underlying these and other type IV modification-dependent restriction systems remain incompletely understood.

### (c) *V. cholerae* Shedu system of West Africa–South America strains

The second defence system encoded in the VSP-II variant of the WASA 7PET lineage belongs to the Shedu group of defence systems [38], and is thus named *V. cholerae* Shedu, or *Vc*SduA ([104]; figure 4A). Types I and II Shedus are single-protein defence systems characterized by a signature DUF4263 domain, which is part of the PD-(D/E)XK nuclease superfamily [38,108]. In type I systems, this domain is preceded by diverse N-terminal regulatory domains, some of which are capable of sensing free DNA ends during phage infection or replication [108,109].

*Vc*SduA was initially tested in *E. coli* using the BASEL phage collection [52] as a screening tool. This assay revealed that the system conferred strong protection against members of the T1 phage-like *Drexlerviridae* family [104]. Tests on its ability to defend against ICP1, ICP2 or ICP3 vibriophages [45] showed no phenotypic differences when comparing the wild-type WASA strain to its VSP-II deletion mutant. However, subsequent research expanded the testing of *Vc*SduA-mediated defence in *V. cholerae* to include older vibriophages, originally used for strain typing, as discussed in the accompanying article by Blokesch & Seed [32]. Through this approach, it was demonstrated that phage X29 is significantly targeted by the *Vc*SduA system [104]. Notably, this activity was initially overlooked in our laboratory owing to functional redundancy between the DdmABC system and VcSduA, both of which target X29 in the WASA lineage of *V. cholerae*.

## 5. Chromosomal integron

Another important genomic feature of *V. cholerae* strains is the presence of a chromosomal integron (figure 1A), also known as the sedentary chromosomal integron (SCI) [110]. Integrons are genetic elements capable of acquiring new gene cassettes and incorporating them at position one of a cassette array, a process mediated by the integron-specific integrase enzyme [111]. A promoter located upstream of the array ensures that the first cassette is highly expressed. However, as additional cassettes are incorporated, those further from the promoter exhibit progressively lower transcriptional activity [111]. Unlike mobile integrons, which are often associated with the spread of antibiotic resistance genes and typically carry only a limited number of cassettes, SCIs are usually large and contain more than 20 gene cassettes, separated by *attC* sites. The SCI array of the 7PET *V. cholerae* strain N16961 was shown to include at least 179 gene cassettes, each typically accommodating one or, occasionally, more than one ORFs. This integron, with its approximately 130 kb length, accounts for approximately 3% of the *V. cholerae* genome ([111]; figure 1A).

While the function of most integron cassettes remains unknown, a recent study by Darracq, Littner *et al.* demonstrated that the integron might serve as a reservoir for defence systems [103]. Given that earlier work showed that deletion of the entire integron had no significant impact on *V. cholerae* physiology [112]—consistent with the presumed low or absent expression of most cassettes not located in close proximity to the promoter—the authors of the current study artificially expressed 88 non-redundant cassettes with unknown functions in either *V. cholerae* or *E. coli*. Subsequently, these cassette-expressing strains were tested against a panel of five vibriophages (ICP1, ICP2, ICP3, X29 and phage 24) and 23 coliphages. The analysis revealed that at least 16 of the 179 integron gene cassettes exhibited anti-phage activity, accounting for approximately 10% of all integron cassettes. These novel anti-phage systems were subsequently characterized bioinformatically [103]. A concomitant study revealed a similar phenomenon, identifying bacteriophage resistance integron cassettes on mobile integrons of diverse bacterial species. These authors therefore suggested that mobile integrons may function as cost-efficient and mobile defence islands [113].

Interestingly, an analysis of a broad range of integrons and their cassettes revealed that defence systems were streamlined to meet the small size requirements necessary for successful recombination of integron cassettes [103]. Indeed, even genes encoding type II or type IV restriction systems were significantly reduced in size when carried within integrons compared to those located outside these genetic elements. Larger defence systems, such as CRISPR-Cas or BREX, which are encoded by more than two genes, were notably absent from integrons [103].

In the study by Darracq, Littner *et al.*, the production of the defence systems was achieved through artificial expression of the respective gene cassettes from plasmids, which resulted in anti-phage activity. To investigate whether natural expression could phenocopy this finding, the authors cloned one of the defence systems (Divona) into the first position of the integron, which confirmed their anti-phage activity. Furthermore, artificial induction of the integrase gene resulted in cassette shuffling, with cassettes excised from various positions within the integron and re-integrated at the first position, where the highest expression level is assumed to occur. This process was demonstrated at the population level through cassette-specific polymerase chain reaction amplification; however, recombined variants with defence systems positioned at the first location of the integron were not isolated.

Collectively, the authors of this work concluded that chromosomal integrons can shuffle anti-phage cassettes and enable their expression when positioned first in the integron array. Thus, these integrons function as biobanks of anti-phage systems. However, while the finding is remarkable, comparative genomics of numerous 7PET *V. cholerae* isolates revealed that the integron neither shuffled its cassettes nor acquired new cassettes at the front positions throughout the ongoing seventh pandemic (since its onset in 1961). This raises an important question: do the newly identified defence systems provide any benefit to the 7PET lineage of *V. cholerae*, given that they might not be expressed at their integron loci and also do not appear to move to a more favourable position closer to the promoter? At this point, it is tempting to speculate that other defence systems within pandemic *V. cholerae*, such as those outlined above, may interfere with the natural function of the integron, preventing cassette acquisition and/or shuffling under natural conditions and restricting these processes to artificially induced integrase expression in laboratory settings. Alternatively, the acquisition of cassettes at the first position of the integron array might be driven by processes related to the environmental lifestyle of *V. cholerae*, rather than the host-adapted niche of 7PET strains. Future research is therefore needed to address these different hypotheses and to determine whether the phenomenon of cassette conservation and positional stability is unique to 7PET strains and sixth pandemic classical strains, which exhibit similarly stable integron patterns [103], or whether it is also observed in environmental, non-pandemic strains. Indeed, integrons in these *V. cholerae* strains differ significantly from those of the 7PET lineage (for a comparison between the 7PET strain A1552 and the environmental strain SA5Y, see [36]) and also exhibit diversity among themselves. This was evident when we analysed long-read-based genome sequences from a set of 15 environmental *V. cholerae* strains [114] originally isolated from the California coast [115]. However, unlike the repeated sampling of patients over the past 60+ years for the 7PET lineage, there is a lack of spatial and temporal data regarding the integron dynamics of non-pandemic *V. cholerae* isolates.

A very recent study by Getz *et al.* reported a similar phenomenon in *V. parahaemolyticus*, identifying its chromosomal integron as a hotspot for anti-phage defence genes. Among 57 tested integron cassettes in *V. parahaemolyticus* strain RIMD 2210633, nine conferred defence when artificially expressed in either the native host or *E. coli* [116].

The study also investigated the rarity of cassette shuffling and whether internal integron promoters might exist that drive defence gene expression. Analysis of publicly available RNA-seq data showed that many integron cassettes, including four defence loci, were induced in a quorum-sensing (QS) mutant mimicking high cell density. RNA-seq further confirmed higher expression of several integron genes during mid-exponential compared to early-exponential phase in this organism [116]. Consistent with this finding, one identified defence system conferred strong protection against vibriophage NL2 from its native integron locus when bacteria were pre-grown in soft agar to high cell density before phage challenge [116]. Whether QS-dependent, integron promoter-independent defence expression is a common feature or an exception remains to be investigated for additional defence systems and other integrons, including the SCI of 7PET *V. cholerae*.

## 6. Discussion and future directions

Recent research has identified several phage defence systems in 7PET *V. cholerae* that are encoded on the conserved MGEs VPI-2, VSP-I and VSP-II. Unfortunately, research on these conserved defence systems within their native *V. cholerae* host remains somewhat limited owing to the scarcity of available phages. Historically, phage isolation was often conducted using pandemic strains as bait (see, for instance [45]), which largely selected against phages sensitive to the core defence systems discussed above. As a result, studying these phage defence systems often necessitates the artificial overexpression of the systems in heterologous hosts, such as *E. coli*, for which large phage collections are available. The establishment of a dedicated *V. cholerae* phage collection, akin to the BASEL coliphage collection [52], would be an invaluable tool for advancing research on these common defence systems. Such a resource would also probably facilitate further discoveries of defence systems in the 7PET lineage of *V. cholerae*.

Studying variable defence systems that are not common to all 7PET strains, on the other hand, can be readily conducted using available phages, such as the ICP phages. As discussed in the accompanying article by Blokesch & Seed, these studies provide valuable insights into the ecology and evolution of defence systems and their host bacteria. Moreover, they facilitate the investigation of defence mechanisms under native expression conditions. When sampled repeatedly over time, such research offers a deeper understanding of the ongoing arms race between 7PET *V. cholerae* and its phages, particularly in patient-derived samples from endemic regions. Indeed, the ability to study phage counter-defence mechanisms is often only possible when co-evolving bacteria-phage pairs are analysed.

Anti-plasmid systems, on the other hand, can be readily studied in 7PET *V. cholerae*, as demonstrated for the T1RM and DdmDE systems encoded on VPI-2, as well as DdmABC encoded on VSP-II [39,40]. Interestingly, the DdmDE and DdmABC defence systems exhibit synergy in 7PET strains. Under the tested conditions, the two systems rapidly eliminated the *V. cholerae* plasmid pSa5Y through degradation processes and by inducing a growth-impaired fitness disadvantage mediated by DdmABC [39,53]. This synergy may help reduce the risk of auto-immunity by depending on the low expression levels required for their combined functionality.

Apparent cooperation between a defence system and other functions encoded by a MGE has also been observed for the CBASS system. Specifically, it was shown that the triggering of the CBASS system in 7PET *V. cholerae* by SMX antifolates could be reversed through the heterologous expression of the resistance gene *sul2* [87]. Interestingly, since the transition from the seventh pandemic wave 1 to wave 2 (around 1978−1984), the majority of 7PET strains have harboured SXT-like ICE elements that confer resistance to antifolates [3,117–119]. These resistance genes probably mitigate the sensitivity of 7PET strains to antifolate antibiotics, thereby ensuring the functionality of the CBASS system under natural conditions. Notably, the most recent 7PET isolates from Yemen exhibit a 10 kb deletion in their ICE elements, including the *sul2* gene responsible for sulfonamide resistance [119]. However, these strains have recently acquired a multidrug resistance plasmid carrying the *sul1* gene, which also confers sulfonamide resistance [57]. This finding highlights once again the intricate interplay between mobile genetic elements within *V. cholerae*.

However, this also highlights another outstanding question in the field: how do such large deletions in MGEs occur, and why are they subsequently selected for? For instance, aside from the 10 kb deletion in ICE elements observed in recent 7PET isolates from Yemen [119], the O139 lineage of 7PET strains also features a large deletion in VPI-2 (figure 2). While purely speculative at this point, two factors may have contributed to the selection of this deletion in the O139 lineage. First, several phages endemic to the regions where the O139 outbreak first occurred (e.g. around the Bay of Bengal), such as ICP1, ICP3, and X29, use the O1 antigen as their receptor. However, O139 strains underwent serogroup conversion by acquiring the O139 antigen cluster at the expense of losing the O1 cluster (figure 1) [27–29]. This probably reduced the selective pressure exerted by many contemporary phages that specifically targeted O1 7PET strains, thereby rendering the ‘defence region’ of VPI-2 potentially less essential. Second, O139 strains acquired SXT-like ICEs, which provided them with additional defence systems [44], as also discussed by Blokesch & Seed in the accompanying article. One such system is BREX [44], a known defence system that includes an epigenetic modifier of the bacterial genome [120]. This system could account for the newly identified *m6A:GTTRAAG:6* DNA modification observed in O139 isolates MO10, MO45 and MO3, as noted above. These methylation marks may render the T1RM system dispensable in terms of self versus non-self DNA recognition and cellular defence.

Lastly, several more recent 7PET strains, such as those isolated in the DRC in 2011 [43] and Bangladeshi isolates from 2018 [97], carry a large approximately 24.5 kb deletion in their integron island. This deletion includes approximately 30% of the newly identified defence systems of 7PET *V. cholerae* [103], namely Taranis, Damona, Lugos, Cernunnos and Brigantia. Additionally, the Bangladeshi isolates [97] also lack the Belenos system. Given that these systems are assumed to be silent at their respective integron positions, their recent loss may not be surprising. However, what advantage this deletion provided to the 7PET lineage, especially given these strains’ spatially separated distribution in recent years, remains another open question in the field.

Collectively, the recent flourishing of the bacterial defence field, marked by the discovery of numerous novel defence systems, has also significantly impacted *V. cholerae* research. By integrating epidemiological data, comparative genomics, and molecular studies aimed at elucidating the mechanisms of these novel defence systems, new hypotheses have emerged. These hypotheses explore how the transmission of pandemic *V. cholerae* has been influenced by antibiotic resistance genes, phages, and MGEs, probably contributing to the sustained success of 7PET *V. cholerae* as a major human pathogen.

**Addendum added during proofing**: Three studies were recently preprinted that further describe the evolution and mechanistic aspects of the DdmABC plasmid/phage defence system and other Lamassu homologues. These studies provided evidence that, upon activation of the defence system, the LmuA effector protein is liberated and assembles into a homo-tetramer, which in turn activates the Cap4 nuclease domain [121–123].

## Data Availability

This article has no additional data.
